# Wheat yield estimation using remote sensing data based on machine learning approaches

**DOI:** 10.3389/fpls.2022.1090970

**Published:** 2022-12-23

**Authors:** Enhui Cheng, Bing Zhang, Dailiang Peng, Liheng Zhong, Le Yu, Yao Liu, Chenchao Xiao, Cunjun Li, Xiaoyi Li, Yue Chen, Huichun Ye, Hongye Wang, Ruyi Yu, Jinkang Hu, Songlin Yang

**Affiliations:** ^1^ 1Key Laboratory of Digital Earth Science, Aerospace Information Research Institute, Chinese Academy of Sciences, Beijing, China; ^2^ College of Resource and Environment, University of Chinese Academy of Sciences, Beijing, China; ^3^ International Research Center of Big Data for Sustainable Development Goals, Beijing, China; ^4^ Ant Group, Beijing, China; ^5^ Ministry of Education Key Laboratory for Earth System Modeling, Department of Earth System Science, Institute for Global Change Studies, Tsinghua University, Beijing, China; ^6^ Land Satellite Remote Sensing Application Center, Ministry of Natural Resources of China, Beijing, China; ^7^ Information Technology Research Center, Beijing Academy of Agriculture and Forestry Sciences, Beijing, China; ^8^ Aerospace ShuWei High Tech. Co., Ltd., Beijing, China; ^9^ Cultivated Land Quality Monitoring and Protection center, Ministry of Agriculture and Rural Affairs, Beijing, China

**Keywords:** band selection, deep learning, google earth engine (GEE), hyperspectral, winter wheat, yield estimation

## Abstract

Accurate predictions of wheat yields are essential to farmers’production plans and to the international trade in wheat. However, only poor approximations of the productivity of wheat crops in China can be obtained using traditional linear regression models based on vegetation indices and observations of the yield. In this study, Sentinel-2 (multispectral data) and ZY-1 02D (hyperspectral data) were used together with 15709 gridded yield data (with a resolution of 5 m × 5 m) to predict the winter wheat yield. These estimates were based on four mainstream data-driven approaches: Long Short-Term Memory (LSTM), Random Forest (RF), Gradient Boosting Decision Tree (GBDT), and Support Vector Regression (SVR). The method that gave the best estimate of the winter wheat yield was determined, and the accuracy of the estimates based on multispectral and hyperspectral data were compared. The results showed that the LSTM model, for which the RMSE of the estimates was 0.201 t/ha, performed better than the RF (RMSE = 0.260 t/ha), GBDT (RMSE = 0.306 t/ha), and SVR (RMSE = 0.489 t/ha) methods. The estimates based on the ZY-1 02D hyperspectral data were more accurate than those based on the 30-m Sentinel-2 data: RMSE = 0.237 t/ha for the ZY-1 02D data, which is about a 5% improvement on the RSME of 0.307 t/ha for the 30-m Sentinel-2 data. However, the 10-m Sentinel-2 data performed even better, giving an RMSE of 0.219 t/ha. In addition, it was found that the greenness vegetation index SR (simple ratio index) outperformed the traditional vegetation indices. The results highlight the potential of the shortwave infrared bands to replace the visible and near-infrared bands for predicting crop yields Our study demonstrates the advantages of the deep learning method LSTM over machine learning methods in terms of its ability to make accurate estimates of the winter wheat yield.

## 1 Introduction

Wheat is one of the most important food crops in China and has the greatest cultivation area and total production among all cereal crops. It has been predicted that world’s total wheat yield will increase by 17% by 2030 due to global warming (Jägermeyr et al., 2021). Therefore, using scientific methods to study the various parameters of wheat growth is very important to ensuring the stability of the country’s wheat market (Weiss et al., 2020). Accurate forecasts of wheat production are of vital importance to farmers’ production plans, the international wheat trade, and import/export plans, and make a direct contribution to the development of China’s wheat market, especially in the context of the COVID-19 pandemic (Mawani and Li, 2020).

Traditional agricultural yield forecasting methods mainly include agronomic forecasting methods (Feng and Wu, 2006), crop-growth models (Thorp et al., 2008), and meteorological statistical methods (Betbeder et al., 2016), and these are used to establish crop yield models based on different perspectives. However, these methods not only consume a lot of manpower and material resources, there are also spatial and temporal gaps in the results. Since 2000, satellite remote sensing technology has played an important role in related fields such as resource surveys (Mitchell, 2021), urban planning (Guo et al., 2019), agricultural development (Qiao et al., 2021), and national security (Zhang et al., 2022). The use of satellite remote sensing has become an effective way of making yield predictions due to its advantages of simple data acquisition, low cost, efficiency, wide spatial coverage, and short operating cycles (Peng et al., 2014; Zhang et al., 2019; Wang et al., 2020).

Vegetation indices (VIs) have been widely used to predict crop yields over the past few decades (Jin et al., 2017; Kamir et al., 2020). In most such studies, indices such as the Normalized Difference Vegetation Index (NDVI) and Enhanced Vegetation Index (EVI) which are based on visible and near-infrared bands (Peng et al., 2010; Cunha and Silva, 2020) are used. However, these vegetation indices mainly reflect the greenness of vegetation and cannot fully capture environmental stresses on crops (Zhang et al., 2021). This means that the role of other vegetation indices such as the Normalized Difference Water Index (NDWI) (Gao, 1996), which reflects the crop water content, and the Red Edge Position Index (REP), which is sensitive to changes in chlorophyll concentration, should be also considered when making yield estimates. In our study, four different types of vegetation indices reflecting crop growth status were used as described above.

Both broadband multispectral data and narrowband hyperspectral data can be used to calculate spectral VIs, but the former are limited and prone to oversaturation where vegetation cover is high (Jiang and Huete, 2010) and thus have difficulty reflecting changes in biophysical and chemical parameters. Narrow hyperspectral bands are more sensitive to crop growth changes than multispectral broad bands (Sellami et al., 2022), but there have been fewer quantitative studies involving the former compared to the latter. Based on narrowband data, the hyperspectral vegetation index (HVI) can fully describe the changes in biophysical and chemical parameters that occur as crops grow, which is important to improving the accuracy of yield estimates made by models (Xiao et al., 2022). Therefore, an increasing number of hyperspectral vegetation indices have been applied to the prediction of crop parameters including the crop yield (Yang et al., 2021), leaf area index (Xing et al., 2013), and nitrogen content (Ma et al., 2022). Further, hyperspectral data typically require sophisticated data mining and filtering techniques given the large number of bands and low signal-to-noise ratio (Marshall et al., 2022). In previous studies, hyperspectral band selection methods include band-by-band combination method (Xing et al., 2013), Optimum Index Factor (Kong et al., 2022), the successive projection algorithm. For example, based on PRISMA hyperspectral images and Sentinel-2 multispectral images, Marshall et al. (2022) used three separate models based on Two-band Vegetation Indices (TBVIs), Random Forest (RF), and Partial Least Squares Regression(PLSR) to estimate the yield of four different crops and revealed the potential complementarity of hyperspectral image PRISMA in predicting crop biomass and yield. However, most studies use only visible wavelengths (Zhang et al., 2018), there are few reported attempts at directly evaluating the potential of Shortwave infrared bands (1000-2500 nm) in crop yield prediction; and investigating the spectral information captured within full waveband range remains unexplored for yield prediction.

The construction of linear regression models linking vegetation indices or climatic variables that track the evolution of crop canopy spectral reflectance patterns over the growing season and yields is the traditional method of estimating yields (He and He, 2013). However, although the calculations may be simple, the relationships involved are not simply linear, and these methods do not capture yield variations well. In the last five years, with the advent of the big data era, conditions have been created for machine learning methods (Zhang et al., 2021), and more and more computer-dependent machine learning models have been applied to crop yield estimation, usually outperforming traditional linear regression. Deep learning (DL) is an advanced Machine Learning (ML) method that uses multiple, stacked nonlinear layers, at each of which the original input data can be transformed into a higher and more abstract representation (Cai et al., 2019), such as Long Short-Term Memory (LSTM), Deep Neural Network (DNN), Convolutional Neural Network (CNN), and Recurrent Neural Network (RNN), have produced definitively higher accuracies across various regression and classification tasks (LeCun et al., 2015). The main advantage of using deep learning techniques in agricultural applications is that the data are hierarchically and incrementally trained with high-level features, eliminating the need to generalize the output. Deep learning models are thus becoming a powerful tool for predicting the yields of various crops (Haider et al., 2019; Sharma et al., 2020; Tian et al., 2021). For example, Zhang et al. (2021) found the LSTM deep learning algorithm outperformed the two other machine learning models in estimating maize yields in China. Huang et al. (2022) developed a Dual-Stream deep-learning neural network model for improving county-level winter wheat yield estimates in China and achieved an average R^2^ of 0.79. Han et al. (2022) integrated an attention-based deep learning framework and the SAFY-V model for winter wheat yield estimation using time series SAR and optical data. Xie and Huang (2021) found the estimated yields from LSTM, 1-D CNN, RF correlated strongly with statistical yields, and the LSTM model achieved the highest estimation accuracies for wheat yields at the site, municipal and county levels. However, the application of ML and DL to yield estimation is still in its infancy, especially in China.

In most studies, yield data are obtained from plot-based manual surveys or consist of county-level regional yields that need to be collected from official statistics websites for larger areas (Sun et al., 2020b). In our study, the yield data used were based on the grid scale (with a resolution of 5 m × 5 m), and accurate measurements were made using specialist instruments at harvest time. These data were more suitable for use as labels to be trained and validated by the models. The number of sample points (15709) was sufficient to allow proper training of the DL and ML models.In this study, using this large number of sampled data together with 30-m ZY-1 02D hyperspectral imagery and 10- and 30-m Sentinel-2 multispectral remote sensing imagery, we established four data-driven models – LSTM (Long Short-Term Memory), RF (Random Forest), SVR (Support Vector Regression), and GBDT (Gradient Boosting Decision Tree) – to estimate winter wheat yields.

## 2 Material and methods

### 2.1 Study areas

**Figure 1:**
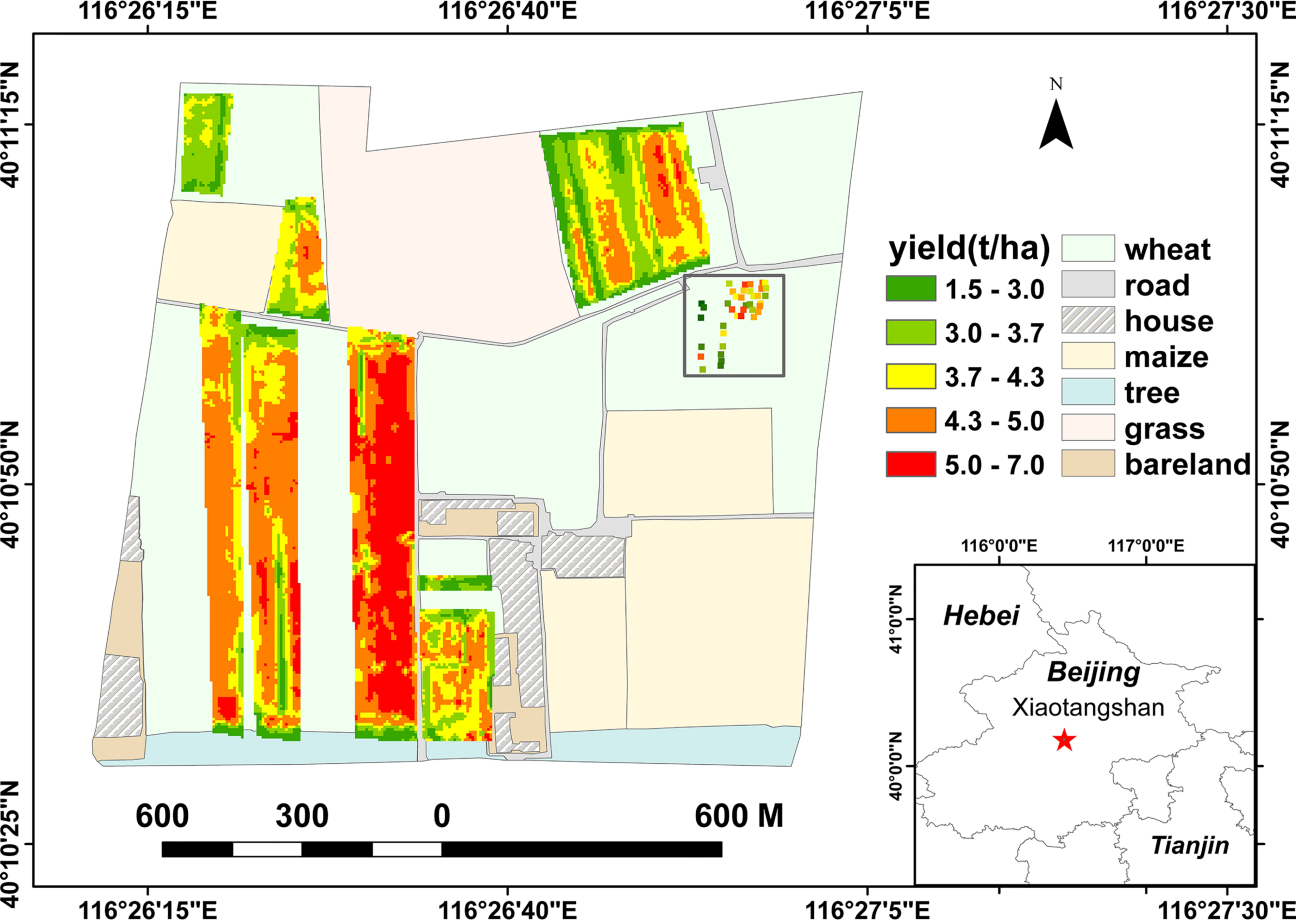
Geographical location and layout of the study area.

Located in the Changping District of Beijing (**Figure 1**), Xiaotangshan National Precision Agriculture Demonstration Base (40.10°N, 116.26°E; altitude 39 m) has a typical climate of the northern winter wheat zone, with an average of 2506.5 hours of sunshine a year, an average annual temperature of 13.3°C, and an average annual rainfall of 563.8 mm. The base is used for high-quality agricultural research area relevant to large irrigated areas and high winter wheat yields.

### 2.2 Datasets and processing

#### 2.2.1 Wheat yield data and auxiliary data

(1) Yield data: From 2020 to 2021, as part of the key project ‘Remote sensing inversion of wheat vegetation parameters based on deep learning’, a yield survey was conducted on winter wheat plots at Xiaotangshan. A total of 15709 dry weight yield data were collected. These data were to be used for training wheat yield estimation models; the values collected ranged from 1.39 to 6.75 t/ha and satisfied the amount of variation that was required. Measurements of the yield were also made at 39 sample points on the ground – within the square area in the upper-right corner of the study area shown in **Figure 1**. These data were used to select the hyperspectral bands.

(2) ASD spectral data: Adjustable speed drives (ASDs) are mainly used to measure the reflectance and transmittance of surface sediments, soils, plants, water bodies, and artificial targets in the range 350–2450 nm. Using an ASD, we obtained spectral data for the 39 sample points on April 14, 2021; these data had a spectral resolution of 3 nm in the 350–1000 nm interval and 8 nm in the 1000–2450 nm interval. From these data, we selected the same 166 bands that are contained in ZY-1 02D data and selected the best vegetation index combination through making correlation analysis with the yield of 39 sample points. The result was then migrated to the band selection of the hyperspectral data.

(3) Grouped data experiments: The data that had been acquired at the 39 sample points were divided into several groups based on the seeding density, irrigation rate, fertilization rate, and seeding method; experiments were then conducted on these different seeding groups of data. A correlation analysis between the different variables and the yield was performed in order to provide data on which the planting of wheat crops could be based.

#### 2.2.2 Remote sensing data

(1) Sentinel-2 imagery: Sentinel-2 is an important optical remote sensing satellite of the European Space Agency’s (ESA’s) ‘Copernicus’ satellite series. Sentinel-2 data are used for land monitoring and can provide images of vegetation, soil and water cover, inland waterways and coastal areas. The Sentinel-2 satellite carries a multispectral imager (MSI) that has a swath width of 290 km and orbits at an altitude of 786 km. The data cover 13 spectral bands and have ground resolutions of 10, 20, and 60 m, respectively. Among all satellite data, only Sentinel-2 data contain three bands in the red-edge range, which means that these data are extremely useful for monitoring vegetation health. In this study, three visible bands, one near-infrared band, one shortwave infrared band, and three red-edge bands were used (see **Table 1**). Winter wheat was sown in the study area on October 7, 2020 and harvested on June 16, 2021. A total of 99 Sentinel-2 images from the ‘COPERNICUS/S2_SR’ dataset in the Google earth Engine (GEE) that covered the period from sowing to harvest were used in this study.

**Table 1:** Details of the data used in this study.

Sensor	Band name	Spectral range (nm)	Band number	Bandwidth (nm)	Spatial resolution raw/resample(m)
Sentinel-2	Blue	458–523	1	65	10/30
	Green	543–578	1	35	10/30
	Red	650–680	1	30	10/30
	Red-edge 1	698–713	1	15	20/30
	Red-edge 2	733–748	1	15	20/30
	Red-edge 3	773–793	1	20	20/30
	NIR	785–900	8	115	10/30
	SWIR1	1565–1655	11	90	20
ZY-1 02D	VNIR	396–1040	76	9	30
	SWIR	1006–2501	90	17	30
ASD	VNIR	350–1000	217	3	—
	SWIR	1000–2450	181	8	—

(2)ZY-1 02D imagery: The ZY-1 02D satellite was successfully launched from the Taiyuan Satellite Launch Center on September 12, 2019 and carries a hyperspectral camera (Advanced HyperSpectral Imager, AHSI) with 166 bands. This instrument has a spatial resolution better than 30 m (9 and 17 nm, respectively in the visible–near-infrared and shortwave infrared bands), a swath width of 60 km, an operating cycle of 55 days, and bands whose wavelengths range from 396 to 2501 nm. Four ZY-1 02D hyperspectral images acquired on March 24, March 30, April 8, and May 1, 2021 were selected for use in this study.

### 2.3 Methodology

#### 2.3.1 Feature selection and its importance

##### 2.3.1.1 Selection of the vegetation index

Using spectral information about the amount of chlorophyll and water absorbed or reflected by a crop in specific wavelength bands, information about parameters related to the growth of the crop can be obtained. From our own spectral measurements, we found that there was strong reflectance from the wheat ears at 850 nm (near infrared) and 1800–1900 nm (shortwave infrared) and that the ratio of the red to near-infrared bands effectively reflected the grain quality of the crop and was well correlated with the yield. We thus selected the simple ratio vegetation index SR for use in this study. In addition, we selected the enhanced vegetation index EVI as another index related to vegetation greenness; this index is based on the blue, red, and near-infrared bands. At late maturity, the spectral properties of plants are strongly influenced by the water content and thickness of the leaves. Absorption bands close to 1.4 µm, 1.9 µm, and 2.6 µm are formed by the absorption of water molecules, and distinct reflection peaks are located at 1.6µm and 2.2µm, between the absorption bands.The intensity of these two reflectance peaks is important for detecting the water content of plant leaves, and based on this spectral feature, we chose a vegetation index NDWI, named by GAO in 1996, to study the water content of wheat. The red-edge band is located between an absorption valley and a peak and covers the range from 690 to 730 nm; the leaf reflectance changes abruptly in this interval. The red-edge band is sensitive to changes in chlorophyll content and is the most obvious to use for detecting stress caused by disease in winter wheat (Jiang and Huete, 2010). We therefore also selected the red-edge position index REP, which is based on the red-edge band of Sentinel-2, for use in this study.

In summary, we selected a total of eight bands in the visible red, green, and blue bands, near-infrared, shortwave infrared and red edge, and calculated the following four vegetation indices: Enhanced Vegetation Index (EVI), Normalized Moisture Index (NDWI), Simple Ratio (SR), and Red Edge Normalized Difference Vegetation Index (REP) (**Table 2**).

**Table 2:** The different vegetation indices used in this study.

VI	Equation	Reference
EVI(Enhanced Vegetation Index)	$2.5*\frac{B8-B4}{B8+6*B4-7.5*B2+1}$	Huete et al. (2002)
SR(Simple Ratio)	$\frac{B8}{B4}$	Jordan (1969)
NDWI(Normalized Difference Water Index)	$\frac{B8-B11}{B8+B11}$	Gao (1996)
REP(Red Edge Position Index)	$705+35*\frac{0.5*(B4+B7)-B5}{B6-B5}$	Horler et al. (1983)

B2, B3, and B4: (visible) blue, green, and red bands; B5, B6, and B7: bands within the red edge; B8: near-infrared band (wide); B11: shortwave infrared band

##### 2.3.1.2 Data preprocessing and calculation of vegetation indices

We called Sentinel-2 data from the Google Earth Engine (GEE) and filtered out all the images in which the cloud cover was greater than 30%. After that, using the GEE, we calculated the mean value of each selected vegetation index in one-month steps for the period October 2020 to June 2021 and constructed a sequence of the mean monthly values. The four ZY-1 02D scenes were first preprocessed in ENVI5.3 – the preprocessing steps included orthorectification, geometric correction, and atmospheric correction. The processed data were then uploaded to the GEE platform for the feature calculation. All bands of both types of imagery were resampled using nearest-neighbor interpolation to the spatial resolution required for our experiments.

##### 2.3.1.3 Feature importance

**Figure 2:**
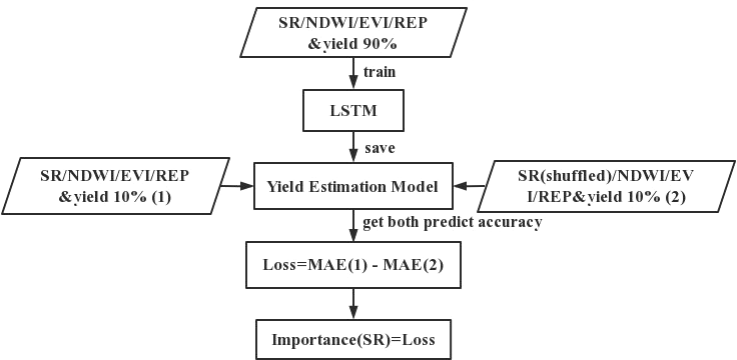
Flowchart of the PFI method.

In recent years, neural networks have been widely used, and they are usually considered black-box models with poor interpretability (Lu et al., 2017). Usually, feature selection mostly takes place at the data-processing stage. This means that parameters such as the number of features need to be set artificially based on experience (Poria. et al, 2015). This introduces a lot of uncertainty, which leads to a loss of learning and generalization ability. To avoid the problem, many researchers have tried using different approaches to incorporate the traditional feature selection process into the networks in order to understand their convolution processes (Krizhevsky et al., 2012; Alain and Bengio, 2018). Various methods of obtaining the feature importance have been proposed: these include Permutation Feature Importance, SHAP Feature Importance, and LOFO Feature Importance, which are universal and can be applied to any model (Breiman, 2001; Fisher et al., 2019). The principle on which the Permutation Feature Importance (PFI) method is based is that the relationship between the features and the true results has been destroyed and that the model prediction error increases after the replacement of the feature values. The PFI approach provides a global insight into the behavior of the LSTM yield-prediction model, and automatically takes into account all interactions with other features. In contrast to methods that remove certain features, PFI does not require the model to be retrained, thus saving time and computational resources. In addition to this, the use of a subset of features seems intuitive; however, the reduced number of features is meaningless in terms of feature importance since we are interested in the importance of the fixed features of the model. In this study, the four vegetation index feature variables (SR, EVI, NDWI, and REP) were input to the proposed model for training, and the importance of each feature was calculated using the PFI method based on the LSTM neural network that we constructed. The steps used to obtain the importance of the neural network features in this study consisted of the following: train the LSTM neural network model; perform a random shuffle on one vegetation index feature to make it not corresponding to yield at a time and put it to the model for prediction to obtain loss; record the corresponding loss of each shuffled feature column. Taking SR as an example, **Figure 2** show the flow of PFI method.

#### 2.3.2 Hyperspectral band selection

ZY-1 02D data consist of a total of 166 bands: 3 of these bands overlap when the VNIR and SWIR bands are partially spliced, giving 163 effective bands. A statistical approach, the band-by-band combination method, was used to select the bands among the 163 bands that were related to high wheat yields. In a recent study (Zhang et al., 2018), the band-by-band combination method that was used consisted of taking every possible pair of bands to construct vegetation indices based on specific mathematical algorithms, and the vegetation index with the highest correlation coefficient with the winter wheat LAI was selected as the best band combination. In this study, we calculated the values of three types of vegetation index – difference, ratio, and normalized – by arbitrarily combining pairs of bands of ASD data that consisted of the same 163 bands as ZY-1 02D data. An analysis of the correlation between the values of these indices and the winter wheat yield was then performed, and the indices that were most relevant to the winter wheat yield were determined. The above results were then used to calculate the values of three ZY-1 02D features:

*SSI*(*i*,*j*)=*R*_*i*_−*R*_*j*_ (1)


(2) $$RSI(i,j)=\frac{R_{i}}{R_{j}}$$

(3) $$NDSI(i,j)=\frac{\left(R_{i}-R_{j}\right)}{\left(R_{i}+R_{j}\right)}$$

Here *i*, and *j* are labels representing any two bands; *R_i_*, and *R_j_* represent the corresponding band values.

#### 2.3.3 Establishment of the yield prediction models

All of the feature-yield data were randomly divided into two groups in the ratio 9:1, with 90% of the data used for training and 10% of the data used for testing. In order to allow a comprehensive evaluation of the experimental results to be made, three metrics were used: the mean absolute error (MAE), the root mean squared error (RMSE), and the coefficient of determination (R^2^). The model that had the largest value of R^2^ and the smallest values of MAE and RMSE was considered to be the optimal one.

The MAE, the RMSE, and R^2^ were calculated as follows: $MAE(X,h)=\frac{1}{m}\sum_{i=1}^{m}\left|h(x_{i})-y_{i}\right|$ (4)

$RMSE(X,h)=\sqrt{\frac{1}{m}\sum_{i=1}^{m}{\left(h(x_{i})-y_{i}\right)}^{2}}$ (5)


(6) $$R^{2}(X,h)=1-\frac{\sum_{i}{\left(h(x_{i})-y_{i}\right)}^{2}}{\sum_{i}{\left(\bar{y_{i}}-y_{i}\right)}^{2}}$$

Here, *y_i_* is the true value of yield, *h(x_i_)* is the value predicted by the yield estimation model, *m* is the number of sample points, $\bar{y_{i}}$ is the mean value of the yield, and *i* is the i-th sample point.

##### 2.3.3.1 LSTM time-series DL model

The LSTM model used a Recurrent Neural Network (RNN) architecture consisting of an input layer, one or more LSTM layers, and an output layer that could learn time-dependent information to incorporate the crop growth process (Hochreiter and Schmidhuber, 1997). The LSTM layers were composed of LSTM cells. Each cell contained three types of gates: the input gates determined what input information was retained, the forget gates determined how much of the previous information input was retained, and the output gates combined the previous output with the current input to determine the final output. In the neural network that was designed, the vegetation index time-series data were passed through two LSTM layers that consisted of 100 neurons, then through an ReLU activation function and a fully connected layer. A dropout rate of 0.3 and L2 regularization were applied to avoid overfitting and improve the generalization effect. We set lr to be 0.001, batch_size to be 64, and epoch to be 700 to further reduce the risk of overfitting. The calculation process of a basic LSTM unit is as follows:


$$\begin{array}{l} i_{t}=\sigma (W^{ih}h_{t-1}+W^{ix}x_{t}+b^{i})\\ f_{t}=\sigma (W^{fh}h_{t-1}+W^{fx}x_{t}+b^{f})\\ g_{t}=\tanh(W^{gh}h_{t-1}+W^{gx}x_{t}+b^{g})\\ c_{t}=i_{t}⊗g_{t}+f_{t}⊗c_{t-1}\\ o_{t}=\sigma (W^{oh}h_{t-1}+W^{ox}x_{t}+b^{o})\\ h_{t}=o_{t}⊗\tanh(c_{t}) \end{array}$$

*W^ih^*, *W^ix^*, *b^i^*, *W^fh^*, *W^fx^*, *b^f^*, *W^gh^*, *W^gx^*, *b^g^*, *W^oh^*, *W^ox^* and *b°* are model parameters; *g_t_* is the nonlinear transformation for better representing the input *x_t_*; *i_t_*, *f_t_*, *o_t_* are the input gate, forget gate and output gate, respectively; *σ*, *⊗*are the sigmoid function and the element-wise multiplication (i.e., Hadamard product) operation, respectively.

##### 2.3.3.2 RF, GBDT, and SVR ML models

Given that the machine learning models (RF, GBDT, and SVR) could not learn time-series information as the LSTM recurrent neural network could, the data had to be converted from a 4×9 matrix format into a 1×36 vector format before being input.

A Random Forest (RF) is formed by integrating multiple decision trees that are trained on randomly selected samples (Breiman, 2001). In our study, 90% of the samples were used for training and the remaining out-of-bag (OOB) samples were used for error assessment. Optimal parameter tuning was performed; the best combination was found to be a setting of 150 for the number of trees and a value of 200 for the random state parameter.The core algorithm of RF is as follows: $\;\underset{\text{A,s}}{\underset{︸}{\min}}[\underset{C_{1}}{\underset{︸}{\min}}{\sum_{X_{i}\in D_{1}(A,s)}\left(y_{i}-C_{1}\right)}^{2}+\underset{C_{2}}{\underset{︸}{\min}}{\sum_{X_{i}\in D_{2}(A,s)}\left(y_{i}-C_{2}\right)}^{2}]$

Where *C_1_* is the sample output mean of *D_1_* data set, *C_2_* is the sample output mean of *D_2_* data set, *A* is the division feature and *s* are the division point, *y_i_* the i-th sample point.

A Gradient Boosting Decision Tree (GBDT) is an iterative decision tree algorithm that consists of multiple decision trees and which uses the accumulated conclusions of all the trees as the final result; the advantage of the GBDT method is its robustness to outliers. Parameter tuning was performed for the GBDT model, and the best combination was found to be: number of trees = 1250, subsample = 0.6, and learning rate = 0.1. The core algorithm of GBDT is as follows:


$$f(x)=\sum_{i=1}^{m}T(X,\theta_{i})$$

Where *T(X,θ)* is the decision tree, *θ* is the parameter of the decision tree, *m* is the number of trees.

A Support Vector Regression (SVR) model is a tolerant regression model that creates an ‘interval band’ with a spacing of ϵ (the tolerance bias, an empirical value set by hand) on both sides of the linear function and which does not calculate the loss for all samples falling into the interval band. The model is obtained by minimizing the total loss and maximizing the interval. SVR is sensitive to the choice of hyperparameters. We selected the Gaussian kernel function (RBF) as the kernel function. As for the other models, the best parameter settings were found by experiment. It was found that the best combination was a value of 1 × 10^5^ for C and a value of 0.5 for gamma. The core algorithm of SVR is as follows:


$$\underset{w,b}{\min}\frac{1}{2}{\left\|w\right\|}_{2}^{2}+C\sum_{i=1}^{m}\left(\xi_{i}+\overset{\wedge }{\xi_{i}}\right)$$

Where *w,b* are the model parameters, *m* is the number of sample points, *ξ* is the relaxation variable.

All the source codes are available at https://github.com/limitlesszang/yield_prediction.

## 3 Results and discussion

### 3.1 Winter wheat yield predictions obtained using the different models

**Figure 3:**
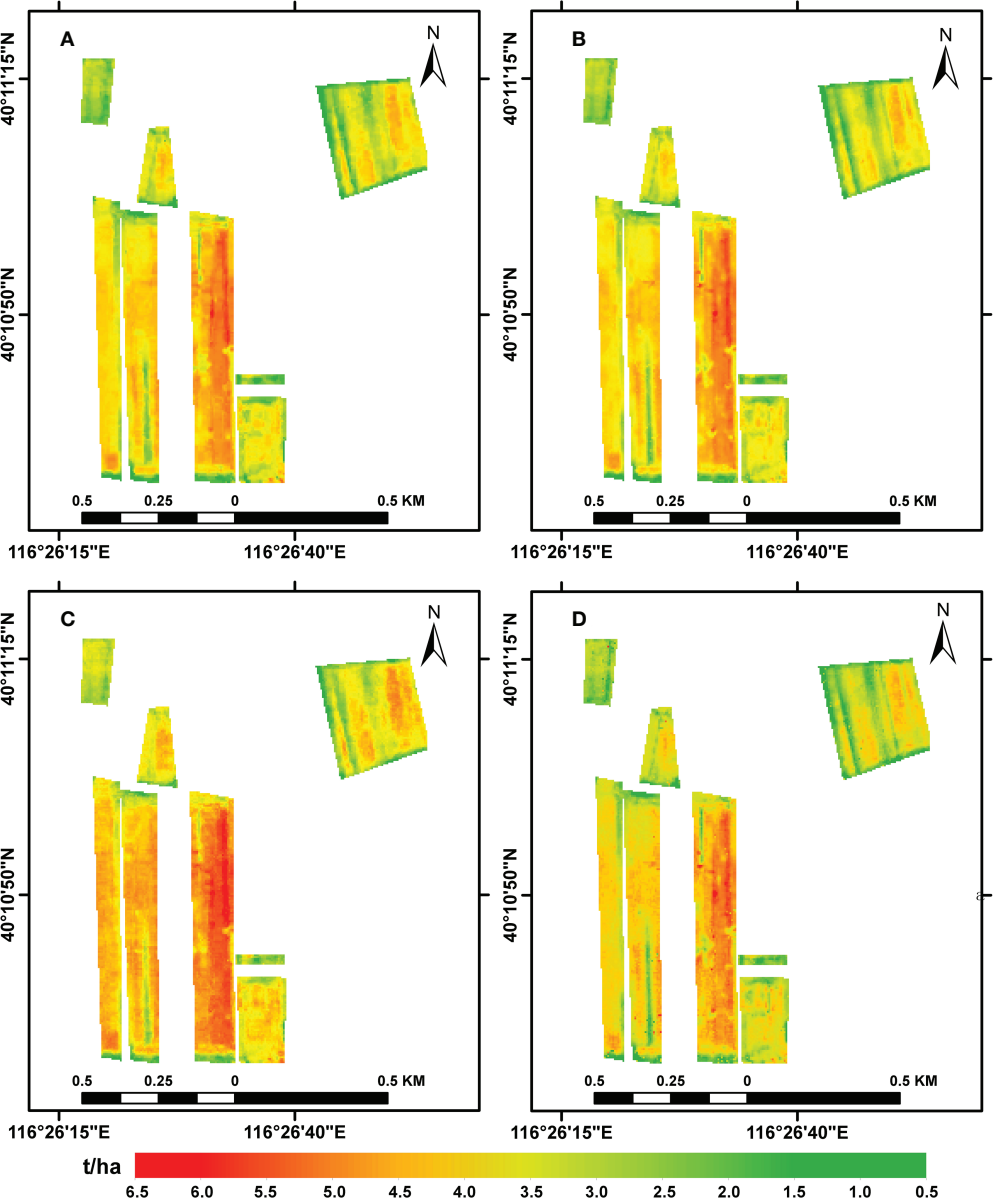
Predictions of winter wheat yields obtained using the **(A)** LSTM, **(B)** RF, **(C)** GBDT, and **(D)** SVR models.

The ability of the four common vegetation indices to predict the winter wheat yield was evaluated using four methods (LSTM, RF, GBDT, and SVR); the results of these predictions are visualized in **Figure 3**. A comparison showed that, of the four models, the LSTM made the best predictions, followed by the RF model. The spatial distribution of the yield predicted by the four models roughly matched the true yield distribution: in each case, the yield was high in the middle of all the plots and low near the edges, a pattern that may have been due to human activities and the presence of trees around the plots. Overall, it was shown that the LSTM, RF, GBDT, and SVR models could be used to make estimates of the winter wheat yield that also reflected the spatial distribution.

**Figure 4:**
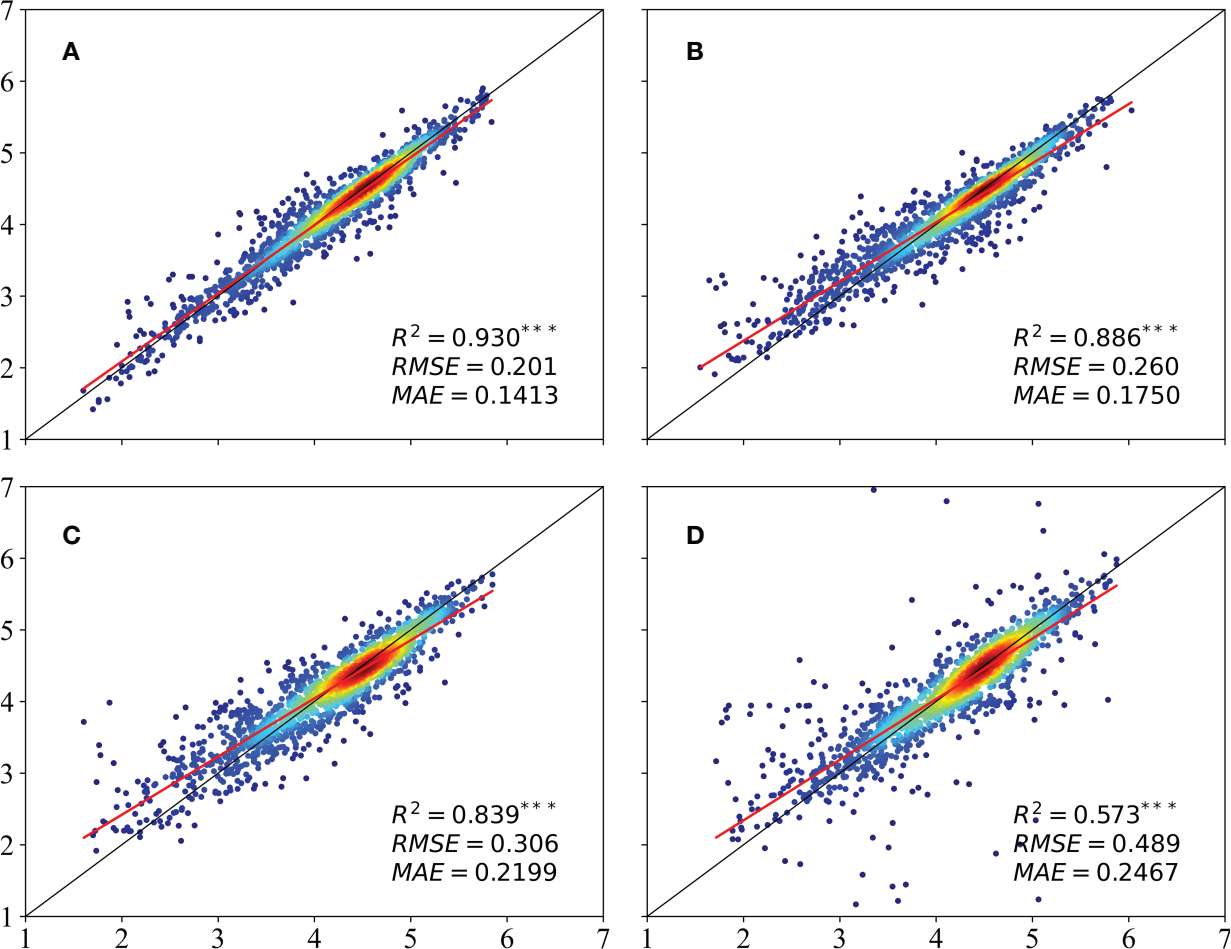
Comparison between the estimates of the winter wheat yield obtained using the **(A)** LSTM, **(B)** RF, **(C)** GBDT, and **(D)** SVR models.

All three metrics (R^2^, MAE, and RMSE) showed that the LSTM model produced the best yield estimates (see **Figure 4**), with a value of 0.93 for R^2^. The neural network architecture of the deep learning model also performed well, giving an R^2^ of 0.886. The values of R^2^ for the GBDT and SVR models were 0.839 and 0.573, respectively. The two tree-based models – RF and GBDT – were able to explain the yield change at least 10% better than the SVR. In contrast, although the SVR technique could effectively solve multiple collinearity problems among independent variables, it only simulates the limited relationship between input variables/features and modeling targets (i.e., grain yield), and is unable to map highly non-linear and complex relationship between variables. As reported in many previous works, deep learning methods are generally considered to be superior when the number of training samples is sufficiently large (Maimaitijiang et al., 2020; Khaki et al., 2021). This is likely due to the fact that DL often exceeds popular machine learning methods when dealing with larger sample size, complex, nonlinear and redundant datasets (LeCun et al., 2015). Our experiments verified that the network containing two LSTM layers could capture more than 90% of the yield information from the input features. Previous research has also demonstrated that LSTM model performed best through several machine learning models in winter wheat prediction. Xie and Huang (2021) demonstrated that the accuracy of the LSTM model was significantly higher than that of the 1-D CNN model due to the better ability of the LSTM model to treat time-series satellite data. However, when the amount of data is limited, the RF model has the advantages of being insensitive to outliers, nonlinearity, serial autocorrelation, and high dimensionality. For example, Cao et al. (2021) found that the performance of RF was not always worse than DL at both the county and field levels. What’s more, although LSTM yielded superior performance over RF methods, the improvement in grain yield prediction accuracy was not substantial (see **Figure 4**), one reason could be the little difference in measured gridded yield data. Future work will examine the ability of more advanced deep learning architectures (e.g., LSTM and its variants) at county scale to extract better information for winter wheat yield prediction.

### 3.2 Estimates of the winter wheat yield based on multispectral and hyperspectral data

**Figure 5:**
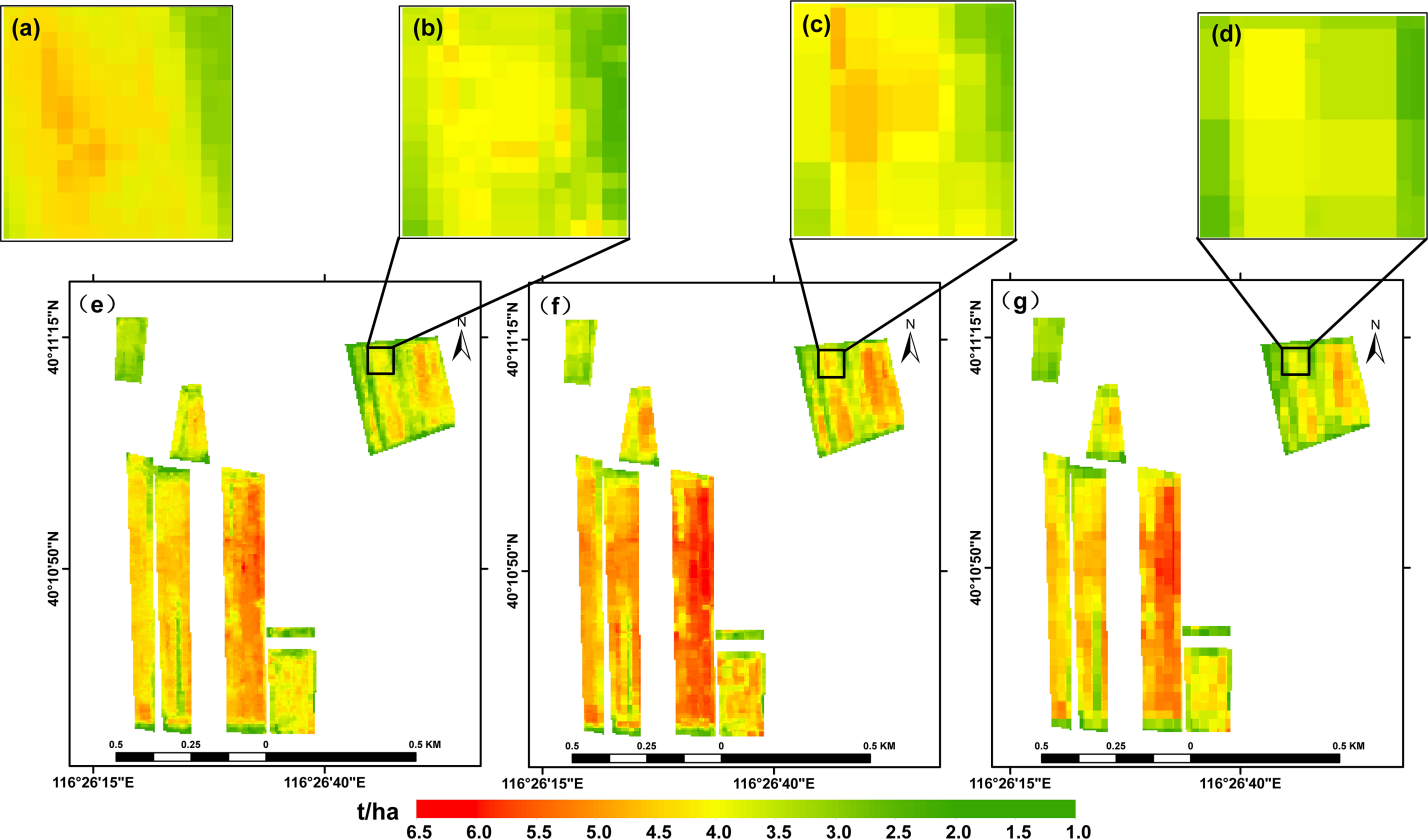
**(A)** Details of the observed distribution of the winter wheat yield. Details of the modeled distribution based on **(B)** 10-m Sentinel-2, **(C)** 30-m ZY-1 02D, and **(D)** 30-m Sentinel-2 data. The modeled yield distribution based on **(E)** 10-m Sentinel-2, **(F)** 30-m ZY-1 02D, and **(G)** 30-m Sentinel-2 data.

The LSTM method was then used to produce estimates of the winter wheat yield based on 30-m ZY-1 02D data, 30-m Sentinel-2 data, and 10-m Sentinel -2 data. The modeling with the ZY-1 02D data used the vegetation indices NDSI, SSI, and RSI designed by ourselves as features, whereas three conventional vegetation indices – NDWI, SR, and EVI – were used with the Sentinel-2 data. The time-series consisting of 20210324, 0330, 0428, and 0501 were input into the LSTM model for training, and predictions of the yield were obtained for the entire study area (see **Figure 5**). It can be seen that, for all three types of data, the yield distribution was correctly modeled and that 10-m Sentinel-2 data best reflect the actual distribution of the yield within the study area. The spatial resolution of the results based on the other two datasets is low; as a result, the corresponding yield distribution maps are coarse and do not reflect the differences in yield between adjacent grid cells.

**Figure 6:**
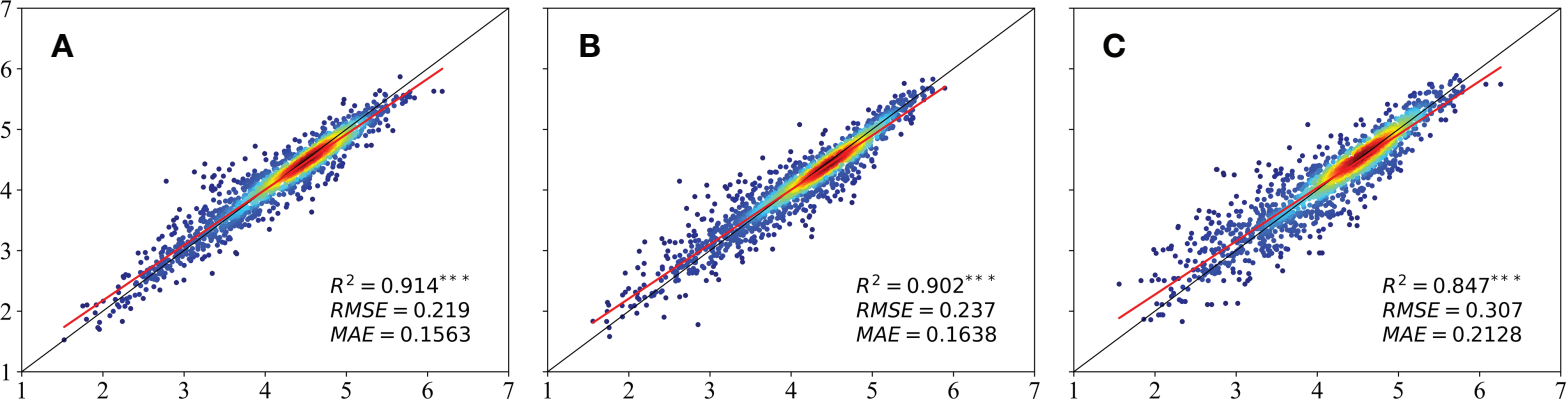
Comparison between estimates of the winter wheat yield based on **(A)** 10-m Sentinel-2, **(B)** 30-m ZY-1 02D, and **(C)** 30-m Sentinel-2 data.

Overall, the estimates based on the 10-m Sentinel-2 data were found to be the most accurate, followed by those based on the 30-m ZY-1 02D data; the estimates based on the 30-m Sentinel-2 were the least accurate (see **Figure 6**). By comparing the results for the datasets with different spatial and spectral resolutions, it was found that the spatial resolution had a greater impact on the winter wheat yield estimates than the spectral resolution: this can be seen from a comparison of the results for the 10-m Sentinel-2 data and the 30-m ZY-1 02D data. The 10-m Sentinel-2 data, which was the dataset with the highest spatial resolution, performed best, capturing 91% of the yield variation. The observed data consisted of gridded data with a spatial resolution of 5 m, and the satellite data with the spatial resolution that was closest to this produced the best estimates of the yield. The result was also recognized in previous studies that hyperspectral PRISMA models was lower than the multispectral Sentinel-2 models (Marshall et al., 2022). However, multispectral bands provide coarser spectral information than hypersectral bands (Yang et al., 2021). A comparison of the results based on the ZY-1 02D and Sentinel-2 data, which have the same spatial resolution, showed that the ZY-1 02D data, which has more spectral bands, performed better, indicating that the features most important to the yield still remained after the band-by-band combination and that the narrow bands could provide relevant and accurate information about the yield. Therefore, Hyperspectral (HS)-Multispectral(MS) fusion paradigm to hyperspectral data is considered to get both advantages of high spatial and spectral resolution. Here, some of the advantages and limitations of applying hyperspectral imaging to estimates of agricultural yields are demonstrated. However, although the mathematical relevance of the hyperspeactral band selection method is maximized, the computational volume is large and the physical meaning is not clear enough, resulting in low applicability (Kong et al., 2022). Further research should be targeted towards to the implementation and evaluation of more applicable band select method in hyperspectral, making the most effective use of hyperspectral band information.

### 3.3 Effect of various spectral bands and vegetation indexes on yield estimation

In the PFI experiment that was conducted, the importance of the four features used in the modeling could be ranked as SR > NDWI > EVI > REP. The shuffled vegetation index SR produced the largest loss of 0.4783, followed by a loss of 0.4492 for the NDWI, 0.2385 for the EVI, and 0.2371 for the REP. A larger loss value indicates a greater contribution to the results. The large SR contribution is due to the high correlation between the ratio of the red band to the NIR wavelength bands and the leaf area index, which is a good measure of the crop growth (Jordan, 1969). The NDWI indicates the amount of biostructural water contained in a crop, so the large contribution made by the NDWI indicates that water has a great influence on the accumulation of organic matter in a crop (Gao, 1996). The EVI, which is a greenness vegetation index, had less effect on the results, which may be related to the instability of the blue band due to residual atmospheric effects. The poor performance of the REP vegetation index in the modeling may be due to the fact that the spatial resolution of the red-edge bands in Sentinel-2 is 20 m, which does not match the spatial resolution of the yield data. However, the vegetation indices we selected were based on anthropogenic experience about spectral information; other vegetation indices such as Green Leaf Area Index (Duchemin et al., 2008), Crop Water Stress Index (Ghaemi et al., 2016) should also be considered. We can combine empirical and statistical methods in the selection of vegetation indices.

In most previous studies, each feature was input to a model individually to retrain the model (Cai et al., 2019). This seems an intuitive approach; however, it is not appropriate if we are interested in the feature importance of the model where all features are trained together instead of one by one. Zhang et al. (2021) evaluated six typical VIs separately for their abilities to predict maize yield using the three approaches. Compared the feature importance rank in Zhang et al. (2021), the advantage of the PFI method that we used in this study is that it outputs the performance of each feature when all of the features are input to the model together, and all interactions with other features are automatically considered. We analyzed the spectral information in combination with the environmental stresses of the crop, and the ranking results reflected the most important factors for wheat growth, so as to provide a reference for practical agricultural management: in conducting winter wheat farming, we need to focus on natural conditions such as tillage density, which is related to the leaf area index, and the amount of irrigation, which is closely related to the water within the winter wheat plant. However, besides PFI, more and more methods have recently been proposed to help users interpret the predictions of complex models, such as a unified framework for interpreting predictions named SHapley Additive exPlanations (Lundberg and Lee, 2017), and further research should be targeted towards to the comparison among multiple feature importance rank methods.

**Figure 7:**
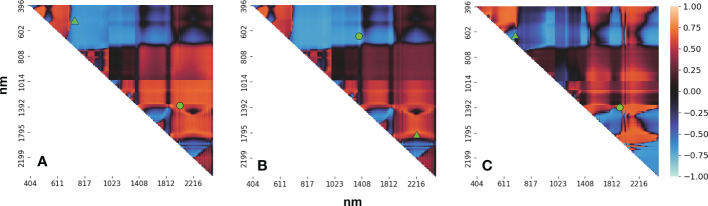
Heatmap showing the correlation between **(A)** the NDSIs, **(B)** RSIs, and **(C)** SSIs and the yield. The green triangles represent the selected band combinations (thickened in **Table 3**); in each case, the green circle represents the other band combination listed in **Table 3**.

From the analysis of the correlation between the different vegetation indices and the yield based on the band-by-band combination method, we determined which bands had the strongest positive and negative correlations with the three different vegetation indices (see **Table 3**). Of the 13203 combinations calculated for the NDSI, the largest correlation with the yield was for the wavelength range 516–765 nm. This lies in the visible and NIR region and was negatively correlated with the yield with a correlation coefficient of –0.7413. For the SSI, the best combination was 671–679 nm, which was negatively correlated with the yield with a correlation coefficient of –0.7559. For the RSI, the strongest correlation was for the combination 1779–2216 nm, which was positively correlated with the yield with a correlation coefficient of 0.7539. The correlation between bands combination and yield is consistent with (Marshall et al., 2022). These three customized vegetation indices were then adopted as three features for input to the hyperspectral data model. The correlation coefficients shown in **Table 3** all pass the significance test of P-value< 0.001. Importantly, from the values of the correlation between the vegetation indexes and the yield (see **Figure 7**), it was found that the combinations of visible and NIR bands were negatively correlated with the yield, whereas there was a positive correlation between the shortwave infrared narrowband combinations and the yield. And can be seen from **Table 3**, the difference between the most positively correlated shortwave infrared narrowband combination and the most negatively correlated visible–NIR band combination is almost negligible (between 0.5% and 5%), which indicates that both combinations can provide equally important information for yield estimates. The combinations of bands around the maximum value in **Figure 7** can almost play the same role as the chosen bands (see **Table 3**) and can also be used to build the yield estimation model when the requirements are not too strict. This conclusion confirmed the results reported in the previous studies choosing the visible and NIR bands to compute VIs (Jin et al., 2017) for yield prediction to various crops. Kong et al. (2022) used the band-by-band combination method between 450-950 nm to construct new vegetation index, and analyzed the correlation of them with LAI. In our work, we expanded band range to full bands following the recommendation of Marshall et al., 2022 to get a comprehensive use of hyperspectral information.

**Table 3:** Band combinations giving the highest correlations with the customized vegetation indices.

	Band combination (nm)	Correlation coefficient	Band combination (nm)	Correlation coefficient
NDSI	1375–1896	0.6946***	516–765	-0.7413***
SSI	1408–1930	0.7506***	671–679	-0.7559***
RSI	671–679	-0.7559***	516–765	-0.7413***

***means the value is significant at the 0.001 level

The high degree of correlation between the visible–NIR band combinations and the yield can be explained by the correlation between the crop growth and the chlorophyll content (Acito et al., 2022), which has also been demonstrated in previous studies (Zhang et al., 2021). However, in most studies, only bands in the range 500 to 900 nm have been used and most other useful bands have been neglected (Zhang et al., 2018; Zhang et al., 2021). In this study, we also found that the shortwave infrared bands between 1000 and 2500 nm had a highly positive correlation with the yield (see **Figure 7**), suggesting that combinations of narrow shortwave infrared bands can provide equally important information to that provided by visible–NIR bands for crop yield estimation and that the use of the two types of information might achieve even better results. In future studies, experiments using the two types of bands should be performed to determine the quantitative relationship with the winter wheat yield.

### 3.4 Analysis of the models and other factors affecting the yield

The results described above (see **Figures 3**, **4**) show that the LSTM model produced significantly better estimates of the winter wheat yield than the other three models; these results are consistent with those found by Zhang et al. (2021) and Lin et al. (2020). Compared with machine learning models, deep learning with complex neural network structures has the advantage that it processes high-dimensional data that reflects the growth and development of crops (Mu et al., 2019; Wang et al., 2020). The LSTM can learn more time-dependent information (Hochreiter and Schmidhuber, 1997). The data input to the models consisted of series of monthly data that can be used to explore various types of changes in crop growth, including the yield and other related crop parameters (Haider et al., 2019; Tian et al., 2021; Wang et al., 2022). The two tree models (RF and GBDT) proved to be better than the SVR model at estimating the yield, which also confirms the results reported in a previous study (Lin et al., 2020). The LSTM neural network provides an effective tool for building new data-driven models for regional yield estimation. Neural network modeling transforms raw input variables into high-level representations through nonlinear activation and squashing functions, which weakens the traceability and interpretability of the LSTM model (Tian et al., 2021). You et al. (2017) add a DGP to Long Short Term Memory (LSTM) network, and outperforming all the competing approaches. Future work will examine the ability of more advanced deep learning architectures (e.g., LSTM and its variants) and more kinds of data (e.g., remote sensing data and climate information) to extract better multimodal information for grain yield prediction. The introduction of an attention mechanism to capture and interpret the contribution of each time node in the time-series data to the models can be considered; in combination with knowledge of the crop phenological period, this could be used to make estimates of pre-production early yields. In subsequent studies, the use of transfer learning methods to improve the scalability of the model could also be tried; this would be similar to a method of predicting winter wheat FVC using deep transfer learning (Yu et al., 2022).

**Figure 8:**
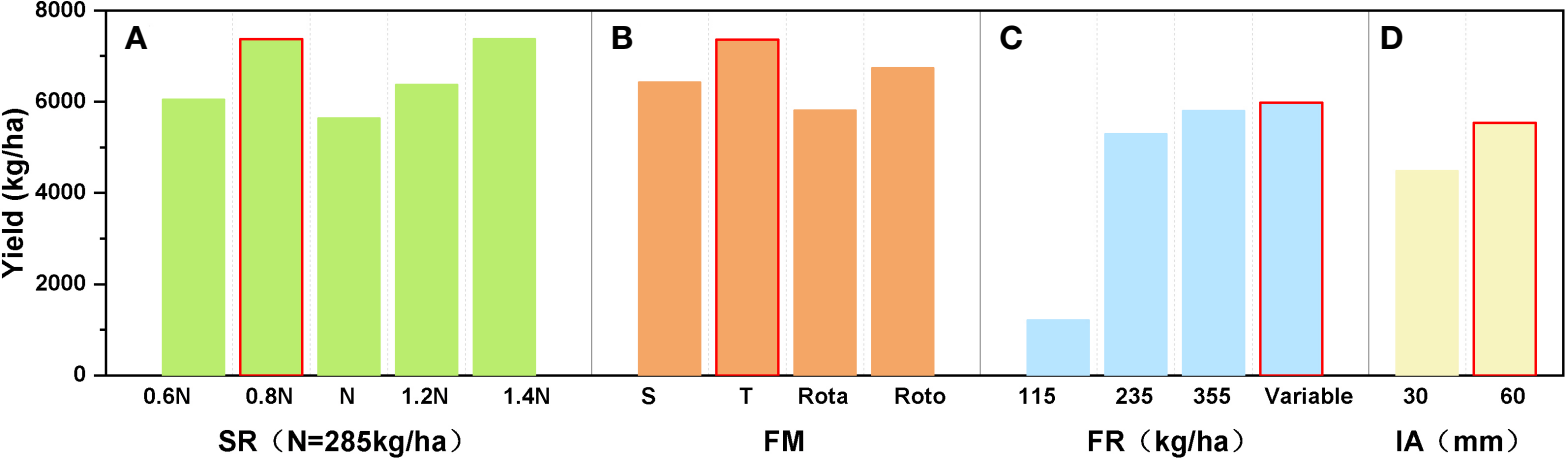
The winter wheat yield plotted against different planting management variables: **(A)** seeding rate, **(B)** farming method (Subsoiling-Tillage-Rotatillage-Rototilling), **(C)** the rate of fertilizer application, and **(D)** the amount of irrigation.

Experiments were then performed in which the Sowing, cultivation and irrigation were varied. It was found that the winter wheat accumulated the most organic matter when the seeding rate was set at 225 kg/ha (0.8 times the conventional sowing rate), rotational tillage was adopted, variable amounts of fertilizer were used, and the amount of irrigation was set at 60 mm (see **Figure 8**). It was also found that a high sowing density leads to a lower yield, probably due to the intense competition between water, fertilizer, and light making the plant less biologically productive and causing lodging (Chen et al., 2022). **Figure 8B** shows that the choice of rotational tillage as the tillage practice can maximize soil fertility: some studies have shown that rotational tillage practices can increase the soil porosity and improve the nutrient quality, thus increasing crop yields (Nie et al., 2015). From **Figure 8C** it can be seen that the use of variable fertilization promotes yield improvement because it meets the nitrogen demand of winter wheat throughout the growing season and allows the crop to maintain a more reasonable canopy structure for photosynthesis even after flowering (Jiang et al., 2015). **Figure 8D** shows that the greater the amount of irrigation, the more water is absorbed by the crop due to osmotic pressure regulation; this increases the soil water storage and improves the drought tolerance of the wheat and the yield. The above meteorological variables are closely related to the crop growth process and directly affect the yield. Currently, as chemical fertilizer prices are rising sharply and many places are experiencing water shortages, advanced studies that will lead to the application of precise amounts of fertilizer and irrigation so that planting costs and environmental pollution can be reduced and high yields of wheat achieved are research priorities (Carberry et al., 2013). Our study quantitatively explored the effect of environmental conditions on the winter wheat yield, and the results provide data that are important to the cultivation of winter wheat in northern China.

Meanwhile, from the above experimental results, it can be seen that crop management statistics have a strong correlation with the crop yield and can be used to indicate yield changes. Management statistics models are included in crop environment models, which can be used in crop yield estimation by establishing a correlation between the crop management statistics and crop yields (Guarin and Asseng, 2022). The most commonly used statistical management model is the systemic integrated factor forecasting method developed by Chen (1992). This forecasting method predicts the annual grain yield by building a systematic model between statistical factors (irrigation, fertilizer usage, and mechanical inputs) and the crop yield. Besides management statistics models, crop environment models (Launay and Guerif, 2005) also include agrometeorological models and agronomic yield estimation models – the former use integral regression models based on meteorological factors and yields (O'Neal et al., 2002), and the latter mainly establish relationships between crop growth conditions and crop yield components, thus allowing them to predict crop yields. Predictions of wheat yields based on management statistics, meteorological data, and crop growth conditions can be highly accurate; however, these models do not apply to large areas and the values of the parameters are difficult to determine. Using satellite remote sensing, crop information can be acquired repeatedly over large areas at a relatively low cost. The combination of remote sensing data with crop growth dynamics models to predict yields has shown promise, and several studies (Cao et al., 2020; Zhang et al., 2021; Beyene et al., 2022) have shown that combining remote sensing and other factors can improve the accuracy of yield estimates. The previous studies demonstrate the tremendouspotential of remote sensing data-based crop yield prediction when employing a multimodal data fusion and deep neural network approach. Maimaitijiang et al. (2020) verified that multimodal data fusion yielded superior performance for yield prediction over single sensor data, regardless of modeling methods. Therefore, in addition to being adaptable to different remote sensing data-VIs, within-field, multi-field, and regional applications require grain yield models to cope with variation and heterogeneity in space caused by differences in soil, irrigation, fertilization and other field conditions that affect plant growth (Maimaitijiang et al., 2020). For example, Su et al (2017) integrated geographical data from the weather station in China and the SVR method to estimate crop growth at various stages. In subsequent research, we plan to collect multiple types of data, including meteorological data and crop management statistics, and combine these with remote sensing data to produced more accurate winter wheat yield estimates.

However, there remain challenges to fully understanding changes in winter wheat yields that arise from a lack of understanding of the mechanisms involved or a lack of data. If fused or integrated data with a high temporal, spatial, and spectral resolution (Cao et al., 2020) can be obtained, transferring our proposed model to a larger study area can be considered. In this study, we found that an increase in either the spectral or spatial resolution leads to an increase in the estimation accuracy (**Figure 4**). Acito et al. (2022) deals with the problem of improving the spatial resolution of hyperspectral data from the PRISMA mission and provides a superresolved image with a spatial resolution of 10 m and the same spectral resolution as the PRISMA hyperspectral sensor. In future, following the work by Acito et al. (2022), In future, we also hope to use fused ZY-1 02D and Sentinel-2 data for yield estimation to explore how temporally, spectrally, and spatially rich data perform, in a similar way to how the fusion of ZY-1 02D and multispectral data has been used for land classification (Sun et al., 2020a). Compared with yield estimation models on county scale (Sun et al., 2020b), the yield estimation models that we developed in this study all apply at the pixel scale and may be less applicable at the larger scale of experimental fields. In subsequent studies, we will collect yield data from a large number of counties and cities, compare agricultural fields in their natural state with small experimental fields under human management, and explore the performance of the yield estimation models over spatially heterogeneous large plots. Furthermore, the approach can be tested for different crop types at different development stages and environmental conditions to evaluate the robustness.

## 4 Conclusion

In this paper, based on Sentinel-2 and ZY-1 02D remote sensing imagery and using the LSTM, RF, GBDT, SVR machine learning methods, we aimed to find the most suitable model, data source, and combination of spectral bands for making estimates of winter wheat yields. It was found that, of these four models, the LSTM model outperformed the SVR, RF, and GBDT models in learning the temporal relationship between the satellite data and the winter wheat yield, giving a value of R^2^ of 0.93. After band selection, the 30-m ZY-1 02D hyperspectral data produced better results than the 30-m multispectral Sentinel-2 data and captured 5% more of the yield variation. However, the most accurate yield estimates were obtained using the data with the highest resolution – the 10-m Sentinel-2 data –for which R^2^ was 0.91. In addition, it was found that the greenness vegetation index, SR, had the greatest effect on the yield estimates, followed by the water index, NDWI. For the hyperspectral data, the combinations of visible and NIR bands were usually negatively correlated with the yield, whereas the linear combinations of narrow shortwave infrared bands were mostly positively correlated with the yield. Our results also show the strong correlation between crop management statistics and yield and suggest the combination of management statistics data and remote sensing data.

In future work, we will consider the application of the modeling to a larger study area and evaluate the performance of fused Sentinel-2 and ZY-1 02D data. Approaches that incorporate migration learning will also be considered.

## Data availability statement

The raw data supporting the conclusions of this article will be made available by the authors, without undue reservation.

## Author contributions

The experiment were mainly conceived and designed by BZ and DP. YL and CX processed the satellite data. EC, LZ, LY, CL, XL, YC, HY, HW, RY, JH and SY performed the experiments. BZ, DP and EC analyzed the data. The algorithm development were mainly accomplished by BZ, DP and EC. EC wrote the manuscript and DP made very significant revisions. BZ helped perform the analysis with constructive discussions. All authors contributed to the article and approved the submitted version.
